# Exploring the Effect of 1-MCP Treatment on the Post-Harvest Quality and Electronic Nose Characteristics of ‘Jizaohong’ Apricots

**DOI:** 10.3390/ijms26104820

**Published:** 2025-05-17

**Authors:** Zhikun Liu, Xuefeng Chen, Chenjuan Jing, Duan Wang, Jingang He, Jianfang Hu, Xiaohong Wu

**Affiliations:** 1 Shijiazhuang Institute of Pomology, Hebei Academy of Agriculture and Forestry Sciences, Shijiazhuang 050061, China; liuzhikun1989321@163.com (Z.L.); chenxuefeng881207@163.com (X.C.); jingchenjuan@163.com (C.J.); wangduanxyz@163.com (D.W.); 2 Institute of Biotechnology and Food Science, Hebei Academy of Agriculture and Forestry Sciences, Shijiazhuang 050051, China; hejingang2000@163.com; 3 College of Horticulture, China Agricultural University, Beijing 100193, China

**Keywords:** apricot fruit, 1-MCP, quality, electronic nose, shelf life

## Abstract

Apricots, known for their unique flavor and health advantages, experience external quality deterioration after harvest due to their climacteric characteristics, leading to a decrease in shelf life. This research examines the effects of 1-Methylcyclopropene (1-MCP) application on the post-harvest quality and volatile compound profiles of ‘Jizaohong’ apricots when stored under ambient conditions. After harvesting, apricots underwent treatment with 0.5, 1.0, and 1.5 µL L^−1^ of 1-MCP for a duration of 24 h, subsequently being stored at ambient temperature (20 ± 1 °C). The results demonstrate that 1-MCP treatments reduced decay, respiration rates, and ethylene production, while also preserving fruit firmness and maintaining skin coloration. Furthermore, the application of 1-MCP markedly diminished the emission of volatile compounds in ‘Jizaohong’ apricots, while linear discriminant analysis (LDA) effectively distinguished between the treated fruits and the untreated controls. The correlation analysis revealed a relationship between the response values of the electronic nose and the quality of the fruit, supporting its potential for swift and non-invasive assessment. Among the concentrations evaluated, 1.0 µL L^−1^ 1-MCP demonstrated the highest efficacy in minimizing decay and improving quality, whereas 1.5 µL L^−1^ 1-MCP did not show notable variations in firmness or ethylene suppression. Thus, the application of 1.0 µL L^−1^ 1-MCP after harvest serves as an effective method for preserving the quality of ‘Jizaohong’ apricots and prolonging their shelf life, while also enabling swift, non-invasive evaluations using the electronic nose.

## 1. Introduction

Apricot (*Prunus armeniaca* L.) is an important seasonal fruit in North China, highly valued by consumers due to its unique flavor and rich nutritional content [1]. However, apricots typically exhibit a short shelf life, with post-harvest softening and decay rapidly reducing their commercial value. Each year, fresh fruit losses during storage and preservation can reach between 20% and 30% [2]. Additionally, some producers resort to early harvesting to accelerate market entry; however, prematurely harvested apricots often have a sour taste and insufficient flavor, negatively influencing consumer acceptance and market revenue. To address this issue, the Shijiazhuang Institute of Pomology developed a new apricot variety, ‘Jizaohong’, registered in 2020 (variety number CNA20200104) [3] and globally introduced through the UPOV platform in 2023 [4]. Hebei Province is China’s second-largest apricot-producing region, with a cultivation area of 12,163 hectares and an annual output of 10,693 tonnes in 2023 (Forestry Statistics Management System of Hebei Province, http://lycy.hebei.gov.cn/report/ (accessed on 7 May 2025)). ‘Jizaohong’s early-maturing characteristic (7 days earlier than ‘Sungold’) enables it to capture premium early-season market prices, significantly enhancing growers’ profitability [3]. It has become one of the four major new varieties recommended by the National Forestry and Grassland Administration of China. This variety matures early (ripening in late May in Hebei Province), produces large fruits (63.5 ± 3.3 g), and has a high yield, reddish skin, a sweet–sour taste, and distinctive aroma [3,5]. However, ‘Jizaohong’ also faces post-harvest preservation challenges. Its early maturity and climacteric characteristics trigger intense post-harvest ethylene production, accelerating fruit softening and susceptibility to mechanical damage, thereby increasing decay incidence and reducing commercial value [6]. Given their limited storage resilience and short shelf life, effective post-harvest treatments to delay senescence and maintain fruit quality are essential. Such interventions significantly extend the market availability of ‘Jizaohong’ apricots and enhance producers’ profitability.

1-MCP serves as a novel inhibitor of ethylene receptors, frequently utilized in the preservation of fruits and vegetables after harvest. By inhibiting ethylene production, the ripening process is effectively slowed, which in turn extends the shelf life of the produce. This compound inhibits ethylene production, delays fruit aging, and extends shelf life. Its application spans various fruits and vegetables [7], such as apple [8], peach [9,10], pear [11], banana [12], papaya [13], and apricot [14]. 1-MCP functions by competitively binding to ethylene receptors, thus blocking the ripening processes induced by ethylene and effectively extending fruit storage time [15]. 1-MCP has demonstrated significant efficacy in extending the post-harvest longevity of apples [16]. The application has shown efficacy in decreasing the decay rate of Fallglo tangerines and white Marsh grapefruits at 50–500 µg L^−1^ [17,18]. Li et al. [19] indicate that 1-MCP has the capacity to reduce decay, slow the deterioration of nutritional quality, and suppress physiological and biochemical metabolic processes during storage. It promotes the overexpression of genes involved in ascorbic acid and aldonic acid metabolism, as well as light signaling pathways, thereby enhancing antioxidant activity and decelerating the aging process of fruits [20]. Research by Kirasak et al. [21] further supports that 1-MCP treatment preserves the integrity of organelles in Dendrobium orchids by preventing the collapse of epidermal and mesophyll cells, as well as the disintegration of specific ultrastructures. Moreover, post-bloom applications of salicylic acid on apricot trees, coupled with post-harvest 1-MCP fumigation, have been shown to improve antioxidant capacity while inhibiting ethylene production and respiration [22]. Additionally, Tong et al. [23] observed that the integration of LMF laser microporous film packaging with 1-MCP treatment effectively prolonged the shelf life of black grapes to 60 days. Additionally, apples subjected to a treatment of 1.0 µL L^−1^ of 1-MCP exhibited increased juiciness, enhanced flavor, and superior visual appeal in comparison to those exposed to alternative concentrations [24]. Nevertheless, employing unsuitable concentrations of 1-MCP can hinder the ripening processes of papayas and pears [11,25]. A dose that is too low may not effectively delay aging, whereas a dose that is too high may cause physiological disorders [13]. Therefore, identifying the optimal 1-MCP concentration for different species is crucial to maximizing its benefits and facilitating its application in agricultural production.

The electronic nose simulates human olfaction to deliver objective and rapid assessments of the sensory quality of fruits. This technology enables fast, non-destructive detection of volatile substances and is characterized by rapid detection, ease of operation, and good reproducibility. Consequently, it is widely employed for variety identification, maturity and decay assessment, and shelf life evaluation [12,26,27]. Principal component analysis (PCA) and LDA are utilized to diminish the dimensionality of odor characteristics, facilitating the examination of the impacts of various preservation techniques on fruits. Previous research has demonstrated that the electronic nose can detect significant reductions in the production of volatile gases in ‘Hanfu’ apples treated with 1-MCP, thereby preserving their quality [28]. Although numerous studies have explored the regulatory effects of 1-MCP treatment on the volatile components of fruits such as apricots [25,29], peaches [30], pears [31], and apples [32], it is important to note that the impact of 1-MCP on post-harvest physiology and quality varies significantly among cultivars. Currently, there is a gap in research regarding the effects of 1-MCP on the volatile characteristics of the unique ‘Jizaohong’ apricot, especially concerning the correlation between electronic nose response signals and post-harvest quality indicators. As apricots are climacteric fruits, their flavor quality declines during storage, making them suitable candidates for quality assessment through the electronic nose’s response to their volatile gases.

This study investigates the effects of different concentrations of 1-MCP on the post-harvest quality, respiration intensity, and ethylene release rate of ‘Jizaohong’ apricots. An electronic nose was employed to analyze changes in volatile gases during storage. PCA, LDA, and loading analysis (LA) were utilized to assess the impact of varying concentrations of 1-MCP on the volatile gases of ‘Jizaohong’ apricots. Furthermore, this research explores the relationship between the electronic nose characteristics of ‘Jizaohong’ apricots and their storage quality, with the goal of identifying the optimal concentration of 1-MCP for apricot storage. This research contributes to post-harvest storage preservation techniques, post-harvest quality, flavor evaluation, and non-destructive detection technology using electronic noses.

## 2. Results

### 2.1. Effect of 1-MCP Treatment on the Flesh Percentage of Decay, Respiration Rate, Ethylene Release Rate, Firmness, and Soluble Solid Content (SSC) of ‘Jizaohong’ Apricot

**Figure 1:**
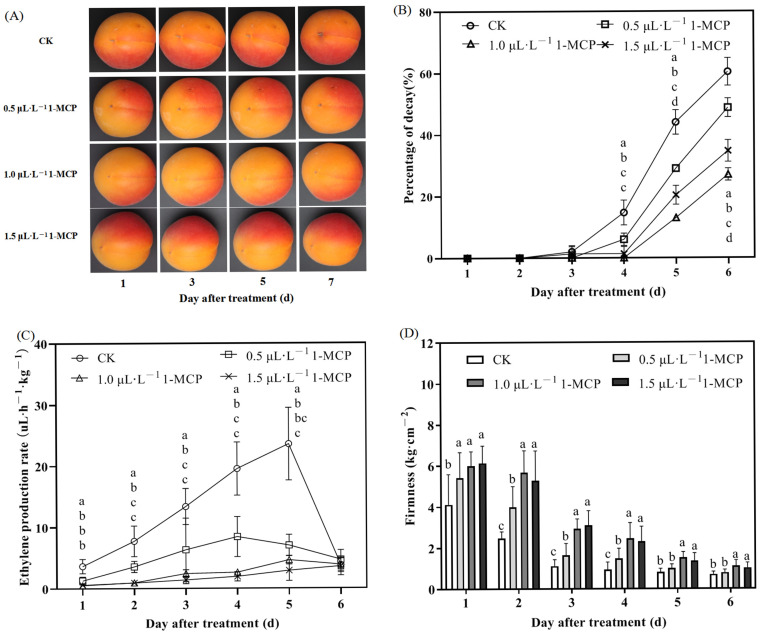
Changes in (**A**) appearance, (**B**) percentage of decay, (**C**) ethylene production rate, and (**D**) firmness of ‘Jizaohong’ apricots treated with 1-MCP during room temperature storage. Different letters denote significant differences among treatments on each day based on Duncan’s multiple range test (*p* < 0.05).

Throughout storage, the apricot percentage of decay progressively increased across all groups (Figure 1B). However, the percentage of decay in the 1-MCP treatment groups was consistently lower. From the fourth day post-treatment, all treated apricots exhibited less decay. Specifically, on the fourth day, 1-MCP treatments at 0.5 µL L^−1^, 1.0 µL L^−1^, and 1.5 µL L^−1^ reduced decay rates by 57.1%, 100%, and 90.5%, respectively. By the sixth day, reductions of 19.6%, 55.2%, and 42.5% were observed in comparison to the control group.

Ethylene, a plant endogenous hormone, promotes fruit ripening and senescence. 1-MCP blocks the irreversible binding of ethylene to its receptor proteins, thus moderating ethylene’s feedback regulatory function. This action reduces ethylene production and delays post-harvest senescence of fruits. As shown in Figure 1C, during the storage process of apricots post-harvest, ethylene release rates initially increased and then decreased. The rates of ethylene release observed across all treatments involving 1-MCP were markedly reduced, showing no significant variation between the concentrations of 1.0 µL L^−1^ and 1.5 µL L^−1^ of 1-MCP. The findings indicate that 1-MCP applications significantly diminish ethylene emission during storage, consequently postponing the processes of fruit ripening and senescence.

Fruit firmness is a key indicator of storage quality, and it tends to decrease as storage time extends. In this experiment, the firmness of apricots in each treatment followed similar trends over time. Compared to untreated apricots, all 1-MCP treatments consistently maintained greater firmness (Figure 1D). With an increase in storage duration, the firmness of the control group fruits exhibited a rapid decline, whereas the firmness reduction in the 1-MCP-treated fruits was more gradual, with all treatments demonstrating elevated firmness levels. During the initial six days post-treatment, the application of 0.5 µL L^−1^ 1-MCP resulted in a reduction in firmness decline by 31.4%, 61.5%, 48.0%, 58.6%, 22.7%, and 11.1%, respectively, indicating notable differences. Nonetheless, a notable difference in firmness was not detected between the 1.0 µL L^−1^ and 1.5 µL L^−1^ 1-MCP treatments, despite the firmness of the 1.0 µL L^−1^ treatment being greater.

Post-harvest changes in respiratory intensity and ethylene release substantially influence storage life, quality, and disease resistance of fruits. Reducing these physiological rates and delaying their peaks can improve commercial value. In this study, 1-MCP treatment effectively reduced respiration and ethylene release rates of ‘Jizaohong’ apricots compared to the control. Respiration rates initially decreased, reaching the lowest level on the third day post-treatment, before rising gradually thereafter; however, rates remained significantly lower compared to untreated controls (Figure S1A). SSC, an important indicator of fruit ripeness and internal quality, influencing fruit flavor and quality evaluation, exhibited a steady increase over storage time without significant differences among treatments (Figure S1B).

### 2.2. Effect of 1-MCP Treatment on the Color Parameters of ‘Jizaohong’ Apricots

**Figure 2:**
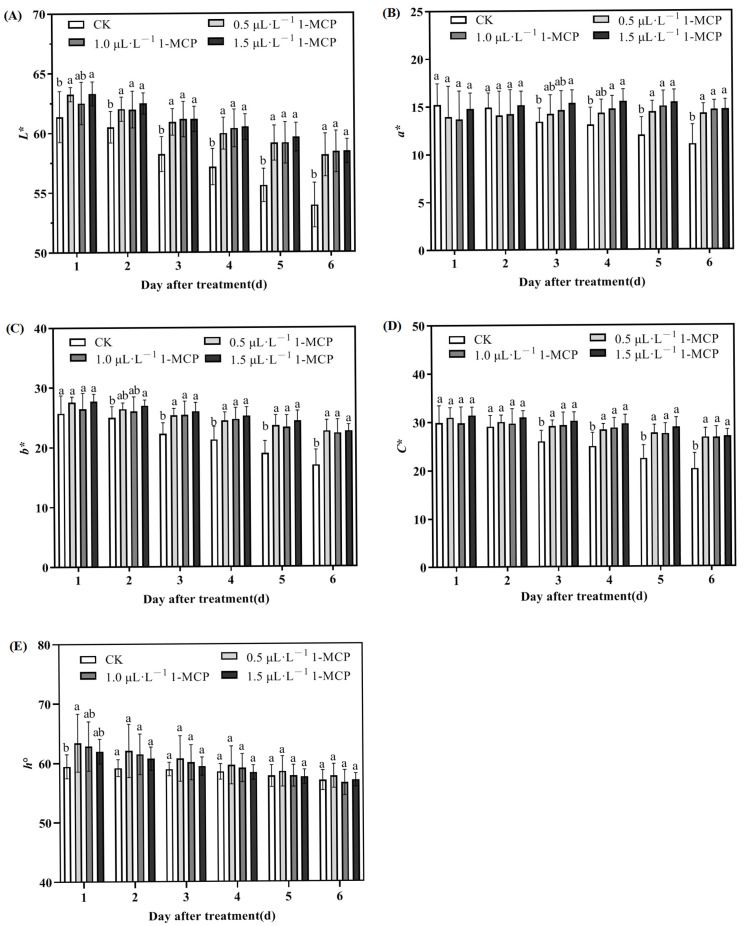
Changes in color parameters of ‘Jizaohong’ apricots during room temperature storage after 1-MCP treatment: (**A**) L*, (**B**) a*, (**C**) b*, (**D**) C*, and (**E**) *h*°. Different letters denote significant differences among treatments on each day based on Duncan’s multiple range test (*p* < 0.05).

The pigmentation of apricots is largely attributed to the presence of various pigments within the fruit’s skin, including chlorophyll (which imparts a green hue), carotenoids (responsible for yellow tones), flavonoids (also contributing yellow), and anthocyanins (which provide red coloration). During the storage period, the L* values exhibited a decline across all treatments, with the control group showing a more pronounced decrease compared to the 1-MCP treatments, a difference that reached statistical significance (Figure 2A). The analysis revealed no notable variations in L* values across the different 1-MCP treatments.

During storage, the a* and b* values for the control group declined rapidly. In contrast, the a* values under the 1-MCP treatments showed a gradual increase, whereas the b* values decreased slowly, with similar trends observed across all three treatments (Figure 2B,C).

The chroma (C*) values decreased over time for all treatments. The control group showed a marked decline, whereas the decline in C* values for the various 1-MCP concentrations was slower and remained significantly higher (Figure 2D). This indicates that 1-MCP treatments inhibit the decline in color intensity of ‘Jizaohong’ apricots. Throughout the storage period, the C* values of fruits subjected to the various 1-MCP treatments consistently exceeded those of the control fruits, exhibiting no significant differences among the treatments. This indicates that all 1-MCP treatments have the potential to postpone the decrease in color intensity of apricots.

The hue angle (*h*°) of all treatments exhibited a decline as the duration of storage increased, revealing comparable trends. On the first day following treatment, the ho values of the 1-MCP treatments were elevated; however, subsequent reductions in *h*° occurred gradually (Figure 2E).

### 2.3. Effect of 1-MCP Treatment on the Electronic Nose Characteristics of ‘Jizaohong’ Apricots

#### 2.3.1. Effect of Different Concentrations of 1-MCP Treatment on the Sensor Response Values of the Electronic Nose

**Figure 3:**
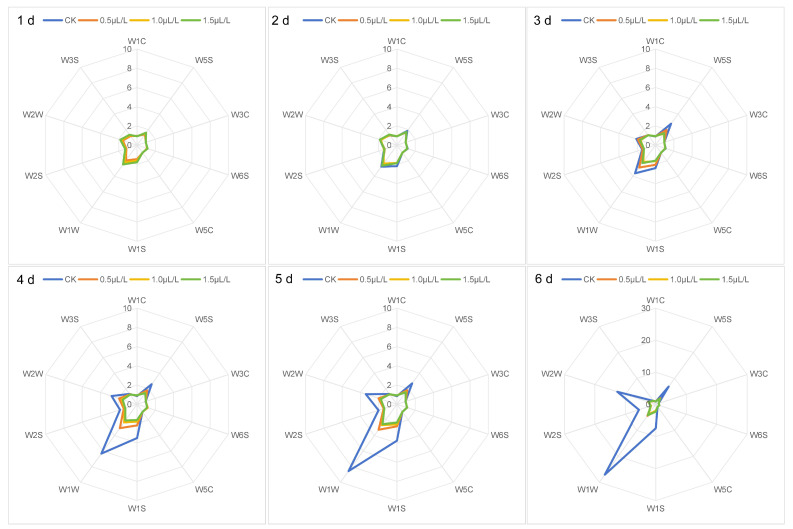
Radar profiles of sensor signal response values for ‘Jizaohong’ apricot volatile gases.

Electronic nose analysis can detect changes in volatile gases emitted by fruits during storage. Figure 3 presents a radar chart of electronic nose data for ‘Jizaohong’ apricots across various storage periods. Throughout storage, the response values of the ten sensors showed similar trends, with each sensor responding differently to the four treatments. On the first and second days post-treatment, the sensor response profiles were consistent, with sensor W1W registering the highest response, followed by W2W and W1S. By the third day following treatment, the control group displayed a notable elevation in the concentrations of four categories of compounds: sulfides and terpenes (W1W), nitrogen oxides (W5S), methyl aromatic compounds (W1S), and organic sulfides and aromatic compounds (W2W), all indicating upward trends and increased levels. On the fourth day, there was a significant rise in the concentrations of alcohols and aldehydic aromatic compounds (W2S). In the 0.5 µL L^−1^ 1-MCP treatment group, the sensors W1W, W2W, W5S, and W1S exhibited an increase on the third day following treatment and maintained a relatively stable condition throughout the storage duration. The odor profiles of the 1.0 µL L^−1^ and 1.5 µL L^−1^ 1-MCP treatment groups exhibited considerable overlap, with only slight variations in sensor response values throughout the storage period. This suggests a similarity in the odors of these groups, as well as a low concentration of volatile components present.

**Figure 4:**
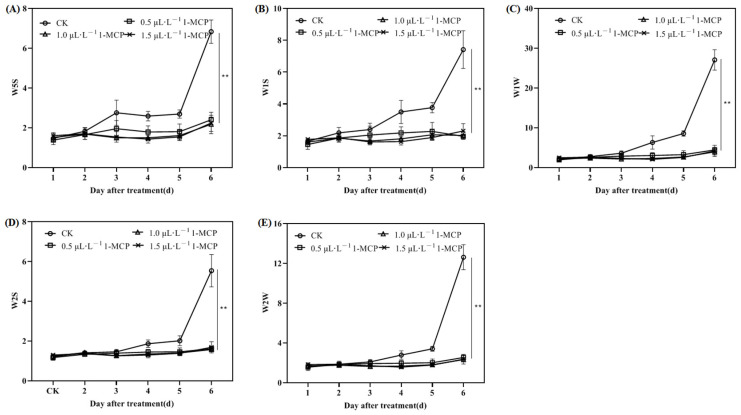
Effect of 1-MCP on the sensor signal response values of volatile gases in ‘Jizaohong’ apricots during room temperature storage ((**A**) W5S, (**B**) W1S, (**C**) W1W, (**D**) W2S and (**E**) W2W). Double asterisks (**) denote extremely significant differences (*p* < 0.01).

To more clearly illustrate the changes in sensor response values, trends of volatile gas responses recorded by sensitive sensors across different treatments during storage are depicted in Figure 4. Sensor responses in the control group gradually increased, with a notable sharp rise beginning on the fifth day after treatment. Treatment with various concentrations of 1-MCP significantly reduced sensor response values (W5S, W1S, W1W, W2S, and W2W). On day 6 post-treatment, the W1W value in the CK group sharply increased to 27.1 ± 2.6 (*p* < 0.01), whereas treatment with 1.0 µL L^−1^ 1-MCP resulted in an 85.2% reduction in response value (Figure 4C). All 1-MCP treatments maintained apricots in a relatively stable state, demonstrating similar trends consistent with radar chart results.

#### 2.3.2. PCA of Electronic Nose Sensor Response Data

**Figure 5:**
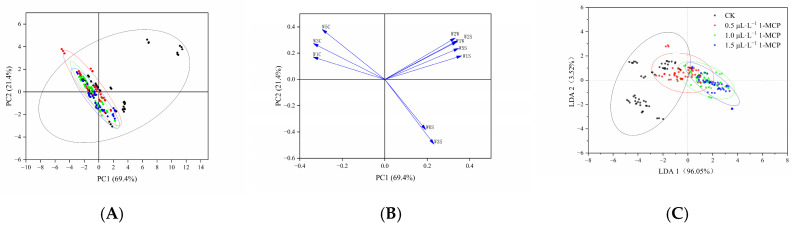
PCA, LA, and LDA of sensor signal response values ((**A**): PCA; (**B**): LA; (**C**): LDA)). The ellipse represents the 95% confidence interval.

PCA was conducted on the changes in volatile gases of ‘Jizaohong’ apricots under different treatments to simplify the electronic nose output data by reducing its dimensionality. PCA was conducted on the response indices of the ten electronic nose sensors (Figure 5A), with principal components chosen according to the criterion of eigenvalues greater than 1. Two primary components were discerned: the first component exhibited an eigenvalue of 6.9 and contributed a variance rate of 69.4%, signifying that it accounted for 69.4% of the total variance, thereby indicating its capacity to encapsulate the majority of the sample variation. The second principal component exhibited an eigenvalue of 2.1, contributing a variance rate of 21.4%, which represents 21.4% of the overall variance. The combined contribution rate of the two primary components reached 90.7%, indicating that they encapsulate the majority of the sample information across all indicators. However, as illustrated in Figure 5A, the areas representing volatile compounds for varying concentrations of 1-MCP treatment exhibit overlap in the PC1 and PC2 plots. This suggests that the regions of volatile substances among the different experimental groups do not fully segregate during the storage of apricots at room temperature. This suggests that this method is not suitable for detecting changes in volatile gases of apricots under different treatments during room temperature storage.

#### 2.3.3. LA of Electronic Nose Sensor Response Values

LA was conducted on the changes in volatile gases of fruits during different storage periods to simplify the electronic nose output data by reducing its dimensionality. Both LA and PCA are based on the same algorithm; however, LA focuses primarily on individual sensors. The greater the distance of a sensor from the origin, the higher its capability to differentiate among samples. This method confirms the contribution rates of each sensor to the samples and identifies the major gases involved.

For the volatile gases of ‘Jizaohong’ apricots, the contribution rates of the first and second principal components were 69.4% and 21.4%, respectively, resulting in a total contribution rate of 90.8%. These results show that PC1 and PC2 capture most of the key sample information. Sensors W2W, W2S, W1W, W5S, and W1S, positioned furthest from the origin on the first principal component, indicate their sensitivity in distinguishing the volatile gases of apricots. Notably, the angles between W2S and W1W, and between W6S and W3S, are particularly small, suggesting significant correlations between these sensors (Figure 5B).

#### 2.3.4. LDA of Electronic Nose Sensor Response Values

The LDA results revealed that the contribution rates for the LDA1 and the LDA2 were 96.1% and 3.6%, respectively, totaling 99.7%. These components predominantly represent the main characteristic information of the samples (Figure 5C). The electronic nose effectively differentiated the 1-MCP treatments from the control group. The volatile compounds present in the 1.0 µL L^−1^ and 1.5 µL L^−1^ 1-MCP treatment groups exhibited a degree of overlap on the coordinate axes with the control group. The characteristics of these two treatment groups were nearly indistinguishable, highlighting their similarity and distinct positioning relative to the control group on the coordinate axes. In contrast, the 0.5 µL L^−1^ 1-MCP treatment exhibited certain similarities with the control group, and there was also a degree of overlap observed between the 1.0 µL L^−1^ and 1.5 µL L^−1^ 1-MCP treatments. In summary, the LDA data exhibited greater concentration, thereby improving the differentiation between treatments (Figure 5C).

### 2.4. Correlation Analysis of Quality, Color Difference, and Electronic Nose Features of ‘Jizaohong’ Apricots

The correlation analysis between the quality, color difference, and electronic nose features of ‘Jizaohong’ apricots demonstrated highly significant correlations among the responses of the five sensors. Firmness exhibited a highly significant negative correlation with the response values of W5S (−0.73), W1S (−0.90), W1W (−0.82), W2S (−0.85), and W2W (−0.77). Both the fruit percentage of decay and SSC showed highly significant positive correlations with the response values of all five sensors. The color difference indicators displayed significant negative correlations with all five sensors, with L*, b*, and C* exhibiting highly significant negative correlations, all with correlation coefficients above 0.70. Overall, the changes in sensor responses partially reflected variations in fruit firmness, SSC, percentage of decay, L*, b*, and C* during room temperature storage of ‘Jizaohong’ apricots (Table 1).

## 3. Discussion

Maintaining post-harvest quality is particularly challenging for apricots due to their climacteric nature and rapid deterioration. The ethylene inhibitor 1-MCP has proven effective in extending fruit shelf life by delaying ripening processes and reducing decay rates in various fruits [33]. Wu et al. [34] reported that 1-MCP treatment effectively decreased decay rates and postponed quality deterioration in blueberries. Similarly, in this study, 1-MCP application significantly reduced apricot decay rates, with the 1.0 µL L^−1^ concentration being most effective. This result aligns with findings by Yuan et al. [35] in blueberries treated with 1-MCP. The likely mechanism is that 1-MCP enhances disease resistance by increasing activity levels of disease-related enzymes such as PPO and POD, thereby reducing fruit decay rates [36].

Apricots are classified as climacteric fruits, and their shelf life is primarily influenced by two critical factors: fruit firmness and ethylene production [37]. Our results demonstrated that 1-MCP application significantly improved apricot firmness compared to the control group. Additionally, apricots treated with 1.0 and 1.5 µL L^−1^ 1-MCP exhibited enhanced firmness throughout storage (Figure 1D). Correlation analysis revealed an extremely significant negative correlation between fruit firmness and decay percentage (r = −0.9, *p* < 0.01; Table 1), indicating that maintaining firmness directly suppresses post-harvest losses. These results align with previous studies on apples, showing that 1-MCP treatment (1.0 µL L^−1^) preserves fruit firmness during storage [24], while ethylene inhibition significantly extends shelf life [38]. Consequently, 1-MCP application effectively delayed firmness reduction in ‘Jizaohong’ apricots. When the 1-MCP concentration exceeded 1.0 µL L^−1^, there were no notable improvements in fruit firmness, corroborating results reported by Zhang et al. [8], and potentially indicating receptor saturation at this threshold concentration. Furthermore, 1-MCP application did not significantly alter SSC in apricots (Figure S1B), demonstrating its selective influence on quality parameters.

Given that 1-MCP functions as a blocker of ethylene receptors, implementing strategies that diminish ethylene buildup post-harvest will prolong the fruit’s shelf life [17]. 1-MCP acts to suppress physiological processes including respiration and ethylene production after harvest, consequently postponing the ripening stage [39,40]. In this study, the application of 1-MCP reduced both respiration and ethylene release rates in apricots throughout the storage period. Treatment with 1.0 µL L^−1^ 1-MCP resulted in an 80.4% reduction (*p* < 0.01) in peak ethylene production (measured on day 5), decreasing from 23.6 ± 5.9 µL h^−1^ kg^−1^ in control fruits to 4.6 ± 0.7 µL kg^−1^ h^−1^ in treated samples. At concentrations of 1.0 µL L^−1^ and 1.5 µL L^−1^, no significant difference was observed in the inhibitory effect of 1-MCP on ethylene release (Figure 1B). 1-MCP exhibits a strong binding affinity for ethylene receptors, interacting with them to induce electron cloud redistribution and receptor conformational changes, thereby disrupting ethylene signal transduction [41]. No significant difference (*p* > 0.05) was observed in ethylene production rates between 1.0 µL L^−1^ (4.6 ± 0.7 µL h^−1^ kg^−1^) and 1.5 µL L^−1^ 1-MCP treatments (2.9 ± 1.7 µL h^−1^ kg^−1^), suggesting receptor binding saturation at 1.0 µL L^−1^ and limited additional effects at higher concentrations. Earlier research has shown that 1-MCP treatment inhibits ethylene production, slows down the ripening processes, and diminishes respiratory activity in ‘Gala’ apples [42,43]. Throughout the storage period, the control group demonstrated a swift rise in ethylene production, which was correlated with heightened respiratory activity, leading to an accumulation of CO_2_ and a reduction in O_2_ levels. The alterations in atmospheric conditions intensified the decline in metabolic processes, ultimately resulting in the decay of ‘Jizaohong’ apricots [44]. The application of 1-MCP resulted in a decrease in the respiration rate of ‘Jizaohong’ apricots, with no significant differences observed among the different 1-MCP treatments. Correlation analysis revealed a significant negative correlation between fruit firmness and both ethylene production (r = −0.77) and respiration rate (r = −0.66) (*p* < 0.01; Table 1), further supporting the critical importance of maintaining fruit firmness. Consequently, under conditions of room temperature storage, the application of 1-MCP can effectively suppress ethylene production and respiration rates, highlighting the role of 1-MCP in postponing the senescence of ‘Jizaohong’ apricots. This aligns with the observed impacts on fruit firmness, exhibiting a greater intensity at 1.0 µL L^−1^, akin to the results reported by Chai et al. [45].

The quality of fruits during post-harvest storage is closely associated with variables like respiration rate and alterations in color. The hue of the fruit’s exterior acts as an essential criterion for assessing its quality, which in turn affects its market worth [46]. In this study, as the duration of storage extended, the L*, a*, and b* values in untreated apricots exhibited a notable decline, signifying the progressive breakdown of red and yellow pigments, which led to a darker fruit skin and diminished brightness. These observations were consistent with visible changes in the apricots’ appearance (Figure 1A). In contrast, 1-MCP treatment effectively inhibited these chromatic alterations (Figure 2). All tested concentrations of 1-MCP resulted in improved color retention and brightness compared with control samples. These findings align with previous reports demonstrating that 1-MCP treatment enhances the retention of green coloration in harvested avocados and substantially reduces yellowing in broccoli compared to untreated controls [47,48]. Furthermore, 1-MCP enhances the pigmentation in peaches by elevating the levels of anthocyanins within the skin, leading to a more pronounced red hue in the fruit [49]. 1-MCP effectively delayed chlorophyll degradation and peel color changes in ‘Jizaohong’ apricots, preserving superior visual quality and marketability.

Factors influencing subjective evaluations of fruit flavor include not only appearance and texture but also aroma and flavor compounds [50]. The volatile compounds responsible for aroma and flavor are produced through metabolism during growth and storage, constituting some of the most crucial sensory attributes of fruits [32]. Earlier research has established that essential components of apricot flavor comprise aldehydes, terpenic compounds, alcohols, esters, and various other volatile substances [51]. Wang et al. [5] demonstrated that terpenic compounds significantly contribute to the characteristic aroma of apricots, with terpenic alcohols serving as crucial indicators for aroma identification; however, excessive synthesis of terpenoids may accelerate fruit quality deterioration. The electronic nose demonstrates high sensitivity to volatile compounds, with minor concentration variations significantly influencing sensor responses [30,52]. This investigation revealed that the sensors most responsive to the volatile gases emitted by ‘Jizaohong’ apricots were W5S, W1S, W1W, W2S, and W2W, with W1W demonstrating the greatest sensitivity. On day 6 post-treatment, the W1W value in the CK group sharply increased to 27.1 ± 2.6 (*p* < 0.01), while treatment with 1.0 µL L^−1^ 1-MCP resulted in an 85.2% reduction in response value (Figure 4C), indicating effective inhibition of terpene production. Correlation analysis revealed that W1W was significantly negatively correlated with fruit hardness (r = −0.82, *p* < 0.01) and significantly positively correlated with percentage of decay (r = 0.76, *p* < 0.01; Table 1). These results suggest that while terpene compounds enhance aroma at optimal levels, their excessive synthesis accelerates quality deterioration of ‘Jizaohong’ apricots. The application of 1-MCP resulted in a delayed response time of these sensors, thereby preserving stability throughout the storage period. At a concentration of 1.5 µL L^−1^, the profiles of volatile gases exhibited remarkable similarity to those observed at 1.0 µL L^−1^, characterized by minimal fluctuations in the response values of each sensor. These findings indicate that 1-MCP not only inhibits the synthesis of certain substances detrimental to apricot quality but also preserves the characteristic volatile composition of ‘Jizaohong’ apricots, including sulfides and terpenes (W1W), nitrogen oxides (W5S), organic sulfides and aromatic compounds (W2W), and methyl aromatic compounds (W1S), preserving their aromatic qualities at levels comparable to those at harvest maturity. This establishes the foundation for the electronic nose’s capacity to differentiate between control and 1-MCP-treated ‘Jizaohong’ apricots based on the volatile gases emitted during storage.

PCA facilitates the analysis of complex multivariate problems through the reduction in data dimensionality. It has been shown to differentiate fresh, cold-stored, and 1-MCP-treated ‘Fuji’ and ‘Hanfu’ apples [28]. Moazzem et al. [53] demonstrated that combining electronic nose technology with PCA can effectively distinguish aroma profiles at various strawberry ripening stages and assess fruit freshness [54]. However, in this study, PCA did not clearly separate CK from 1-MCP-treated groups, resulting in overlapping distribution areas (Figure 5A). This suggests limited effectiveness for tracking 1-MCP-induced changes in ‘Jizaohong’ apricots stored at room temperature. LA based on PCA assessed the relative importance of each sensor to the samples. In this experiment, the sensors W2W, W2S, W1W, W5S, and W1S were farthest from the origin on the first principal component (Figure 5B), indicating their importance during the storage of apricots. In contrast, LDA emphasizes the analysis of distances between samples, projecting sample information into a direction that maximizes separation between groups [27,55]. Zhu et al. [32] applied LDA with an electronic nose to different apple varieties, finding no overlap among them, which improved their ability to distinguish between them. As shown in Figure 5C, the LDA plot reveals four distinct clusters with varying degrees of separation. Notably, there is partial overlap between the control group and the 0.5 µL L^−1^ 1-MCP treatment, while the other two 1-MCP treatment groups exhibit significantly better separation. These results demonstrate that LDA can effectively discriminate aroma profiles of ‘Jizaohong’ apricots treated with 1-MCP concentrations exceeding 1.0 µL L^−1^. Compared to PCA, the LDA shows more tightly clustered data points and superior group differentiation, indicating its enhanced capability for distinguishing between different treatment groups.

Correlation analysis showed that the response values of the sensitive sensors W5S, W1S, W1W, W2S, and W2W were significantly correlated with indicators such as fruit quality and color difference. Electronic nose-based detection analysis can effectively discriminate between 1-MCP-treated and untreated fruits by LDA, while the electronic nose technology is capable of reliably estimating quality parameters such as apricot firmness and peel color. This provides a scientific basis for rapid, non-destructive evaluation of the storage quality of ‘Jizaohong’ apricots. Compared to other concentrations, the 1.0 µL L^−1^ 1-MCP treatment significantly delayed fruit softening, preserved flesh coloration, and maintained the flavor of ‘Jizaohong’ apricots at commercial maturity levels. However, electronic nose technology is primarily limited to macroscopic discrimination of volatile profiles, lacking the capability to precisely identify specific volatile compounds and their concentrations. In subsequent investigations, we propose to integrate GC-MS with metabolomics approaches to systematically elucidate the regulatory effects of 1-MCP on multidimensional fruit quality attributes.

## 4. Materials and Methods

### 4.1. Plant Materials and Treatments

Chinese scientists at the Shijiazhuang Institute of Pomology grew the fruit for the newly created apricot cultivar ‘Jizaohong’. The fruits chosen for this study were consistent in size (62.71 ± 1.60 g), free of any obvious physical damage, and at a similar maturity level (evidenced by red-blushed skin and easy pedicel detachment). Their total soluble solids content was 12.8 ± 0.92% and their firmness levels were 7.29 ± 0.87 kg cm^−2^.

A concentration of 0.5, 1.0, and 1.5 µL L^−1^ of 1-MCP were employed in the trials. Packaging for the apricots was carried out using commercial PE plastic, which had dimensions of 65 cm × 70 cm and a thickness of 0.02 mm. Four groups were given the fruits at random. The groups that received treatment were given doses of 1-MCP ranging from 0.5 µL L^−1^ to 1.5 µL L^−1^, and the containers used for this were 17.6 L cardboard boxes. A separate group received the identical treatment but was kept in an airtight container for 24 h to avoid exposure to 1-MCP. The 1-MCP utilized in the research was SmartFresh^TM^, manufactured by Agrofresh Solutions, Inc. (Philadelphia, PA, USA). It had a 1-MCP level of 0.014% and was packaged in bags of 0.625 g. Five cartons containing about three kilograms of fruit (about forty-eight fruits) were used for each treatment. The fruits were kept at room temperature (20 ± 1 °C) with a relative humidity of (90 ± 10%) after the PE packets were opened, which took about 24 h. Fruit respiration rate, ethylene release rate, electrical nasal response, color parameters, intrinsic quality (firmness, SSC), and other pertinent indices were measured daily.

### 4.2. Firmness and SSC

The GY-1 fruit firmness tester, manufactured by Zhejiang Top Cloud-agri Technology Co., Ltd. (Hangzhou, Zhejiang Province, China), with an indenter diameter of 3.5 mm, was used to measure fruit hardness in kg cm^−2^. The SSC, on the other hand, was measured in percent using a PAL-1 handheld digital sugar meter, manufactured by Atago Co., Ltd. (Tokyo, Japan).

### 4.3. Color Parameter Measurement

A Konica Minolta Co., Ltd. (Tokyo, Japan) CR-400 automatic color difference meter was used to assess the variations in the ‘Jizaohong’ apricot peel’s color. The brightness of the fruit is indicated by the L* value, where higher values indicate more brightness. The change from negative green to positive red is represented by the a* value, while the change from negative blue to positive yellow is measured by the b* value. ΔE* represents the total color difference, with larger values signifying more pronounced color changes in the fruit. Hue angle [*h*° = arctan (b*/a*)] indicates the hue shift. The higher the chroma (C* = (a *^2^ + b *^2^)1/2), the brighter the color intensity [32].

### 4.4. Respiration Rate, Ethylene Production Rate, and Electronic Nose Analyses

Three independent trials comparing treated and untreated fruits were conducted to evaluate respiration. At room temperature (20 °C), respiration chambers were filled with five fruits of known weight and volume for each replicate. The flow-through system was selected for its ability to closely mimic commercial storage conditions. The respiration chambers were 1 L airtight plastic containers with lids, and a single 8 mm diameter hole was drilled into each lid. A silicone hose was connected and sealed to facilitate multiple insertions of the sampling needle.

The experimental groups consisted of control and 1-MCP treatments, with each group having three biological replicates. The respiration rate, ethylene production rate, and electronic nose analyses were measured accordingly.

The HWF-1A CO_2_ infrared analyzer, manufactured by Kexi Instrument Co., Ltd., (Jintan, Jiangsu Province, China) was used to measure the respiration rate (µL kg^−1^ h^−1^) after 0.5 h of individual fruit encapsulation in 1 L jars and subsequent injection of 10 mL of gaseous sample [56].

A gas chromatograph designed for ethylene chromatography, the GC 9790 IIB (Zhejiang Fuli Analytical Instrument Co., Ltd.; Taizhou, Zhejiang Province, China), was used to measure the ethylene production rate in units of µL kg^−1^ h^−1^. After soaking the fruits in a 1 L container for five hours, the gas chromatograph was flushed with 1 milliliter of the sampled gas.

$$\mathrm{Respiration}\;\mathrm{rate}\;(\mathrm{mL}\;{\mathrm{kg}}^{-1}\;h^{-1})\;=\frac{\mathrm{Change}\;\mathrm{in}\;\mathrm{gas}\;\mathrm{concentration}\;\times \;\mathrm{Chamber}\;\mathrm{airspace}\;(\mathrm{mL})}{\mathrm{Fruit}\;\mathrm{weight}\;(\mathrm{kg})\;\times \;\mathrm{Measurement}\;\mathrm{interval}\;(h)}$$

Change in gas concentration = CO_2_(T_1_) − CO_2_(T_0_)/100

Fruit volume = Fruit weight × density

Chamber airspace = Total chamber volume − (Fruit volume + 10 mL *)

* volume of gas sample drawn each time.

With certain tweaks to the usual method, the odor profile of ‘Jizaohong’ apricot fruit was analyzed using a portable PEN 3 electronic nose (Airsense, Schwerin, Germany) [32,46]. A temperature of 25 °C was used for the evaluations. After three hours, we took gas samples from the container and ran them through the electronic nose for analysis. The air cleaning duration was set at 200 s, the injection flow rate was kept at 400 mL min^−1^, and the measurement period was 110 s. The research showed stable G/G0 levels between the 90th and 92nd s. Table A1 shows the characteristic responses of the 10 metal oxide sensors that were installed in the electronic nose.

### 4.5. Statistical Analysis

The use of three biological replicates was standard in all tests and investigations. Excel and GraphPad Prism 9 were used for data organization and charting. SPSS 18.0 for Windows was used to conduct a one-way analysis of variance (ANOVA). To find out if there was a statistically significant difference (*p* < 0.05) between the groups who received 1-MCP treatment and the control group, Duncan’s multiple range tests were used. The digital dataset was examined, categorized, and its dimensionality reduced using Winmuster (Airsense, Schwerin, Germany), which allowed for a PCA of the electronic nasal data. Drawing, PCA, LA, and LDA were all executed using Origin 2022.

## 5. Conclusions

Throughout the storage duration, the application of 1-MCP markedly suppressed the respiration and ethylene emission rates in ‘Jizaohong’ apricots, leading to a significant postponement of their softening process. The intervention exhibited no notable impact on soluble solids, thereby preserving optimal fruit quality and minimizing spoilage rates. Furthermore, the application of 1-MCP treatment effectively maintained the structural integrity of the red pigments in the apricots, ensuring their vibrant appearance and significantly preserving both flavor and color during storage at room temperature. Nonetheless, 1-MCP reduced the electronic nose’s sensitivity to volatile compounds in ‘Jizaohong’ apricots, leading to a slight decrease in the synthesis of volatile substances. In the evaluation of treatments, the application of 1.0 µL L^−1^ 1-MCP demonstrated the highest efficacy in minimizing fruit decay. Additionally, the parameters of firmness and ethylene release rates did not exhibit notable differences when comparing the 1.0 and 1.5 µL L^−1^ treatments. Under these circumstances, the ideal concentration for prolonging storage duration is 1.0 µL L^−1^ of 1-MCP. Consequently, the application of 1.0 µL L^−1^ 1-MCP after harvest serves as a viable method for maintaining the quality of ‘Jizaohong’ apricots and prolonging their shelf life. The application of electronic nose technology can accurately assess quality parameters, including the firmness of fruit and the coloration of its appearance.

## Figures and Tables

**Table 1:** Correlation coefficient between intrinsic quality, color difference, and volatile gas response value of ‘Jizaohong’ apricot.

	Percentage of Decay	Ethylene Production Rate	Respiration Rate	Firmness	SSC	L*	a*	b*	*h*°	C*	ΔE*	W5S	W1S	W1W	W2S	W2W
**Percentage of Decay**	1.00															
**Ethylene Production Rate**	0.55 **	1.00														
**Respiration Rate**	0.65 **	0.52 **	1.00													
**Firmness**	−0.90 **	−0.77 **	−0.66 **	1.00												
**SSC**	0.82 **	0.54 **	0.59 **	−0.86 **	1.00											
L*	−0.87 **	−0.77 **	−0.62 **	0.97 **	−0.88 **	1.00										
a*	−0.23	−0.37	−0.2	0.31	−0.25	0.33	1.00									
b*	−0.85 **	−0.78 **	−0.64 **	0.95 **	−0.88 **	1.00 **	0.34	1.00								
***h*°**	−0.86 **	−0.50 *	−0.65 **	0.85 **	−0.85 **	0.87 **	−0.08	0.87 **	1.00							
C*	−0.81 **	−0.77 **	−0.64 **	0.92 **	−0.87 **	0.97 **	0.45 *	0.98 **	0.80 **	1.00						
ΔE*	0.80 **	0.39	0.52 **	−0.74 **	0.68 **	−0.73 **	0.24	−0.70 **	−0.89 **	−0.59 **	1.00					
**W5S**	0.64 **	0.69 **	0.53 **	−0.73 **	0.59 **	−0.73 **	−0.53 **	−0.73 **	−0.51 *	−0.76 **	0.35	1.00				
**W1S**	0.67 **	0.81 **	0.59 **	−0.80 **	0.69 **	−0.82 **	−0.43 *	−0.82 **	−0.60 **	−0.84 **	0.46 *	0.90 **	1.00			
**W1W**	0.76 **	0.73 **	0.65 **	−0.82 **	0.70 **	−0.81 **	−0.48 *	−0.80 **	−0.62 **	−0.82 **	0.47 *	0.96 **	0.92 **	1.00		
**W2S**	0.78 **	0.71 **	0.69 **	−0.85 **	0.78 **	−0.86 **	−0.43 *	−0.86 **	−0.70 **	−0.88 **	0.53 **	0.91 **	0.92 **	0.95 **	1.00	
**W2W**	0.72 **	0.67 **	0.63 **	−0.77 **	0.63 **	−0.74 **	−0.49 *	−0.73 **	−0.56 **	−0.76 **	0.42 *	0.97 **	0.89 **	0.99 **	0.92 **	1.00

* Significant correlation (*p* < 0.05). ** Extremely significant correlation (*p* < 0.01).

## Data Availability

Data are contained within the article.

## Supplementary Materials

The following supporting information can be downloaded at: https://www.mdpi.com/article/10.3390/ijms26104820/s1.

## Abbreviations

The following abbreviations are used in this manuscript:

PCA	principal component analysis
LA	loading analysis
LDA	linear discriminant analysis
SSC	soluble solid content
1-MCP	1-methylcyclopropene

## Appendix A

**Table A1:** Standard sensor arrays and performance specification in electronic nose PEN3.

Number	Sensor	Performance	Notes
S1	W1C	Aromatic Compounds	Methylbenzene, 10 ppm
S2	W5S	Nitrogen Oxides	NO_2_, 1 ppm
S3	W3C	Ammonia and Aromatic Compounds	Benzene, 10 ppm
S4	W6S	Hydrogen	H_2_, 100 ppm
S5	W5C	Short-chain Aliphatic Compounds	Dimethylmethane, 1 ppm
S6	W1S	Methyl Aromatic Compounds	CH_3_, 100 ppm
S7	W1W	Sulfides and Terpenes	H_2_S, 1 ppm
S8	W2S	Alcohols and Aldehyde Compounds	CO, 100 ppm
S9	W2W	Organic Sulfides and Compounds	H_2_S, 1 ppm
S10	W3S	Long-chain Alkanes	CH_3_, 100 ppm
