# Supplementary material for: Exploring the Effect of 1-MCP Treatment on the Post-Harvest Quality and Electronic Nose Characteristics of ‘Jizaohong’ Apricots
Source: Int J Mol Sci. 2025 May 17;26(10):4820. doi: 10.3390/ijms26104820 (PMC12112520; doi:10.3390/ijms26104820)
Supplement: Supplementary file 1 [file ijms-26-04820-s001.zip › ijms-3514823-supplementary.pdf]

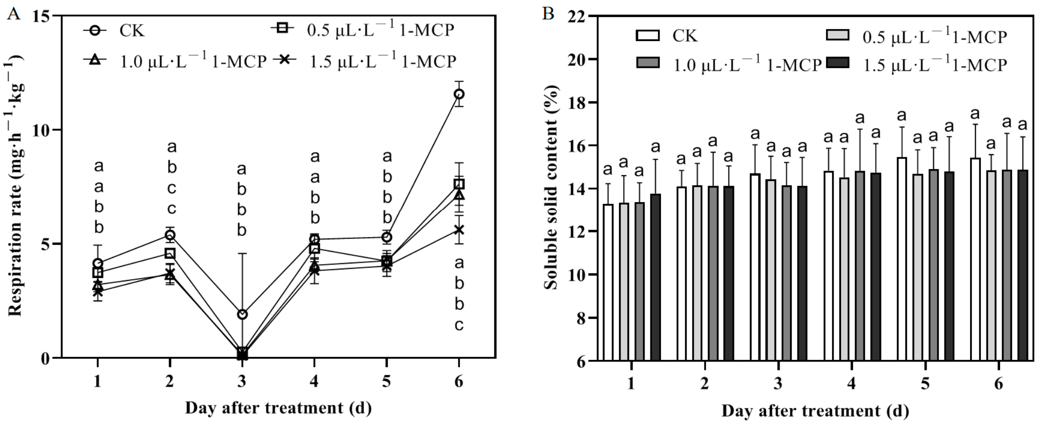

Figure S1. Changes in respiration rate (A) and SSC (B) of 'Jizaohong' apricots treated with 1-MCP during room temperature storage. Different letters denote significant differences among treatments on each day based on Duncan's multiple range test ($P < 0.05$).
